# Editorial: Pathogenic mechanism of porcine viral disease

**DOI:** 10.3389/fvets.2024.1488296

**Published:** 2024-09-17

**Authors:** Mengmeng Zhao, Xuelian Zhang, Lingxue Yu, Weili Kong

**Affiliations:** ^1^Guangdong Provincial Key Laboratory of Animal Molecular Design and Precise Breeding, College of Animal Science and Technology, Foshan University, Foshan, China; ^2^Shanghai Veterinary Research Institute, Chinese Academy of Agricultural Sciences, Shanghai, China; ^3^University of California, San Francisco, San Francisco, CA, United States

**Keywords:** swine viral diseases, PRRSV, pathogenic mechanism, PRV, PEDV

## 1 Introduction

Porcine viral disease is the most serious disease in the pig industry. Bacterial diseases have been widely controlled because of the use of antibiotics. Although some drugs or vaccines have been used in viral diseases, many of them are not effective and the treatment cost is high (1). And many of them stay in the laboratory exploration stage, and it is difficult to conduct large-scale experiments in clinical productions. Exploring the pathogenesis of animal viral diseases is helpful to the pathogenesis research of viral diseases, the rational use of drugs and vaccines, and the development of new vaccines. Nowadays, new viral diseases are constantly emerging in swine diseases, including the recombination and variation of old viral diseases, and the emergence and cross-species transmission of new viruses. Taking China as an example, in recent years, a new variant PRRSV NADC-34 strain appeared in 2014 (2), and the African classical swine fever virus appeared in 2018 (3). The old virus has not been eliminated, and new viruses have appeared, which makes the disease prevention and control of pigs increasingly complicated.

There are many reasons behind it, including the variation of natural climate and the unreasonable abuse of vaccines. Many factors lead to more and more animal diseases.

## 2 Organization of the Research Topic

The theme of this Research Topic mainly discusses the *Pathogenic mechanism of porcine viral disease*. This Research Topic has received 30 manuscripts, 10 manuscripts (two reviews, one opinion, one brief report, and six original articles) were accepted, 20 papers were rejected, the acceptance rate was 33.3%, and two Manuscript Summaries were also received. This Research Topic covers porcine reproductive and respiratory syndrome virus (PRRSV), pseudorabies virus (PRV), and Senecavirus A virus and porcine epidemic diarrhea virus (PEDV). These manuscripts were from nine laboratories in China, Hungary, Britain, and Namibia.

Ren et al. constructed a multiplex qRT-PCR method, which can distinguish PEDV, *B. hyodysenteriae*, and *L. intracellularis*. These three diseases are often shown as mixed infection in clinic diseases. Guo et al. successfully constructed a new infectious clone against the current epidemic PRRSV strain, and successfully saved the virus, laying a foundation for studying the characteristics of the new strain. Senecavirus A (SVA) does great harm to pig industry. Li, Chu et al. made a transcriptome analysis of cells infected by Senecavirus A and found 565 upregulated and 63 downregulated ones, which laid a foundation for revealing the infection mechanism of this virus. Opriessnig et al. developed a weaned piglet intubation model, and all vaccinated pigs showed strong immune responses and maintained protection against PRRSV attack. Xu et al. identified the protein that interacts with the N protein of PEDV through interactive genomics, such as the second-largest subunit of RNA polymerase II (RPB2) and uridine phosphorylase 1 (UPP1), both of which are involved in nucleotide metabolism. Overexpression of RPB2 was observed to significantly promote viral replication, while overexpression of UPP1 was found to inhibit viral replication significantly. The study of the interaction between PEDV N and host proteins provides a theoretical foundation for further exploration of the pathogenic mechanisms and strategies for the prevention and control of PEDV. Li, Liu et al. specifically explored the role and mechanism of RNA recombination in Senecavirus A, suggesting that attention should be paid attention. Jakab et al. demonstrated the development of a tiling amplicon sequencing protocol for the analysis of genome sequence stability in the context of the modified live PRRSV vaccine strain, Porcilis MLV. The results indicated that ARTIC-style protocols can be employed in the evaluation of genomic stability in PRRS MLV strains. Molini et al. detected PRRSV in Africa for the first time, which is type I and may be introduced from Europe. Tan et al. identified the proteins that interacted with the host during the adsorption and invasion of PRV infection, which laid the foundation for further research. Pei et al. summarized that differences in genetic background lead to different biological shapes, including resistance to PRRSV, internal immunological mechanism, mechanism of entering cells, differences in symptoms and lesions after infection, differences in viral load, and differences in non-coding RNA.

## 3 Conclusion

This Research Topic reveals the interaction and replication mechanism of different viruses from many aspects, which is helpful to further reveal the replication mechanism of these viruses and provide theoretical reference for controlling these diseases in the future.
